# Discovery of presumably introduced spiders, *Oedignathascrobiculata* Thorell, 1881 (Araneae, Liocranidae) and *Boagriusqiong* Lin & Li, 2022 (Araneae, Palpimanidae) on Chichi-jima Island, the Ogasawara Islands, Japan

**DOI:** 10.3897/BDJ.12.e121421

**Published:** 2024-04-12

**Authors:** Yuya Suzuki, Yu Hisasue

**Affiliations:** 1 Tokushima Prefectural Museum, Tokushima, Japan Tokushima Prefectural Museum Tokushima Japan; 2 Ogasawara Division of Japan Wildlife Research Center, Tokyo, Japan Ogasawara Division of Japan Wildlife Research Center Tokyo Japan

**Keywords:** alien species, China, fauna, Hainan Island, oceanic islands, Pacific Ocean, Southeast Asia

## Abstract

**Background:**

The spider fauna of the Ogasawara Islands, oceanic islands located 1,000 km south of mainland Japan was comprehensively examined by the National Museum of Nature and Science in 2011, which revealed that approximately 70% of the spider fauna was composed of non-native species. Following the preceding study, however, only descriptions of several new species have been added and no major updates have been made for the overall spider fauna of the Islands.

**New information:**

The faunistic survey of spiders conducted on Chichi-jima Island, the largest island in the Ogasawara Islands in 2023 revealed the presence of two ground-dwelling spider species, *Oedignathascrobiculata* Thorell, 1881 (Araneae, Liocranidae) and *Boagriusqiong* Lin & Li, 2022 (Araneae, Palpimanidae) on the Island. This represents the first record of the two species from Japan, the first record of palpimaid spiders from Japan and the initial documentation of liocranid spiders in the Ogasawara Islands.

## Introduction

**Figure 1.**
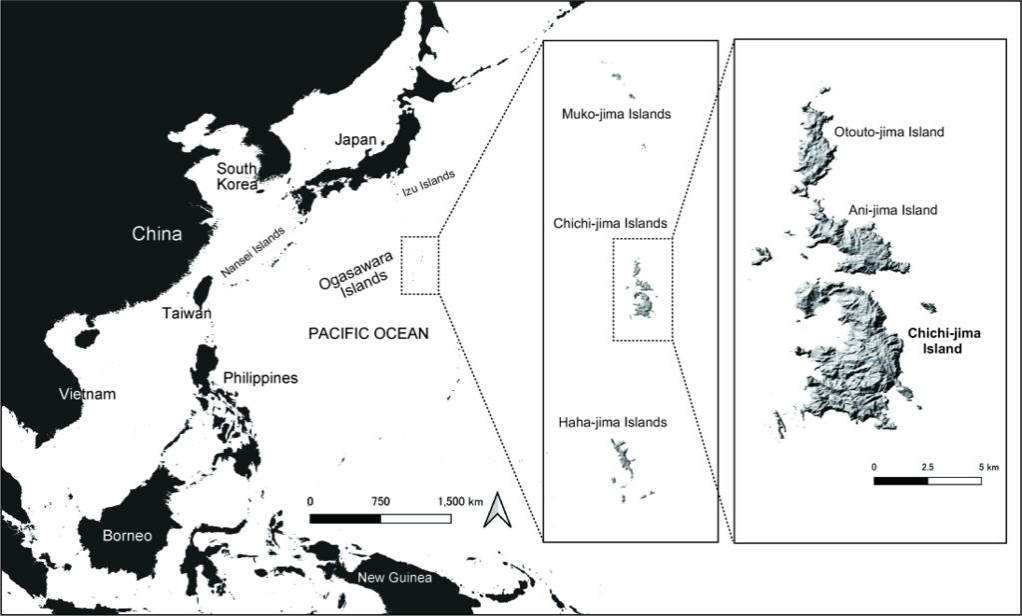
Map showing the locality of Chichi-jima Island, where *Oedignathascrobiculata* and *Boagriusqiong* were recorded.

The Ogasawara Islands are oceanic islands located 1,000 km south of the mainland of Tokyo (Fig. 1). Due to the geographic isolation, the islands’ fauna boasts numerous endemic species (Karube 2004, Shimizu 2010, Chiba 2010). Regarding the scientific value as the typical examples of adaptive radiation on small oceanic islands, the Ogasawara Islands have been designated as a UNESCO World Nature Heritage Site. In recent decades, however, introduction of invasive alien species such as *Anoliscarolinensis* Voigt, 1832 (Reptilia, Squamata, Iguanidae), *Rhinellamarina* (Linnaeus, 1758) (Amphibia, Anura, Bufonidae) and *Platydemusmanokwari* De Beauchamp, 1963 (Platyhelminthes, Tricladida, Geoplanidae) pose a serious threat to the endemic arthropods and molluscs of these Islands (Karube 2005, Ohbayashi 2006, Uchida et al. 2016, Shinobe et al. 2017), which leads to an increase in the importance of frequent monitoring on the fauna of the Ogasawara Islands.

The spider fauna of the Ogasawara Islands were scarcely known until recent decades (Shimomura 1933). Prior to the 1930s, only two spider species were documented, namely, *Pachylomerusmirandus* Kishida, 1921 (Araneae, Halonoproctidae) [a junior synonym of *Conothelefragaria* (Dönitz, 1887), supposed by Ono (2011)] and *Cladothelaboninensis* Kishida, 1928 (Araneae, Gnaphosidae) (Kishida 1921, Kishida 1928). Following several faunistic reports, such as Yaginuma (1970) and Shinkai (1991), the spider fauna underwent its first comprehensive study by Ono (2011). This study, based on specimens deposited in the National Museum of Nature and Science, Tokyo, along with additional field surveys, revealed a total of 81 species belonging to 25 families on the Islands.

As a result of the preceding faunistic survey, it was found that the spider fauna of the Ogasawara Islands was predominantly composed of artificially imported species (70% of the fauna), with a low proportion of natural inhabitants (30%) (Ono 2011). Ono (2011) also noted that some arboreal native salticid spiders were not discovered on Chichi-jima Island during the survey between 2009 and 2010, suggesting possible extinction of these species due to predation pressure by alien predators. Subsequent updates to the fauna were provided by Suguro and Nagano (2015), Baba et al. (2015), Baba and Tanikawa (2018) and Shimano et al. (2018). Currently, the fauna of the Ogasawara Islands recognises approximately 90 species belonging to 25 spider families. However, no comprehensive faunistic data on the spider fauna of the Ogasawara Islands have been reported since Ono's study in 2011.

To update and evaluate the current spider fauna on the Ogasawara Islands, the authors have recently been surveying the spider fauna of various sites on Chichi-jima Island, the largest and most populous island in the Islands. During the survey, we discovered an unknown, 8-eyed, dark-coloured ground-dwelling spider with a scutum on the dorsum of the abdomen, exhibiting characteristics consistent with *Oedignatha* (Araneae, Liocranidae) and a reddish-orange coloured spider resembling members of palpimanids (Araneae, Palpimanidae). Close morphological examination of these specimens revealed that they can be identified as *Oedignathascrobiculata* Thorell, 1881 (Araneae, Liocranidae) and *Boagriusqiong* Lin & Li, 2022, respectively. *Oedignathascrobiculata* is known to be native to South and Southeast Asia and speculated to have been introduced to Madagascar, Seychelles, Reunion and Germany and *Boagriusqiong* has been recorded exclusively from Hainan, China (World Spider Catalog 2024).

As part of our survey of the spider fauna on Chichi-jima Island, this brief report presents the first records of *Oedignathascrobiculata* and *Boagriusqiong* from Japan, based on specimens collected from the Island. Additionally, we provide photographs and the habitat of these species in Chichi-jima Islands and a brief discussion on the presumed introduction of these species from other regions.

## Materials and methods

Specimens were collected from litter layers and preserved in 70% ethanol solution. Photographs of these specimens were taken using a digital camera (habitus: LAOWA 50 mm F/2.8 2X ULTRA MACRO APO; genitalia: Olympus OM-System Zuiko Auto-Macro 38 mm and Olympus Auto 65-116 mm attached to Olympus OM-D E-M1) and stacked using an imaging software (Zerene Stacker; Zerene Systems, Washington, USA). A living image was taken using a Canon EOS 60D with a Canon MP-E 65 mm lens and focus-stacked using Zerene Stacker image stacking software. Habitats were photographed with a digital camera (Olympus Tough TG-5). Internal female genitalia was clarified by 10% potassium hydroxide (KOH) solution. Measurements are given in millimetres. Measurements of the legs are given in the following format: femur + patella + tibia + metatarsus + tarsus = total. Specimens used in this study are deposited in the arthropod collection of the Tokushima Prefectural Museum, Tokushima, Japan (*O.scrobiculata*: TKPM-AR 3191, 3193; *B.qiong*: TKPM-AR 3219).

Terminology of the genital morphology is in accordance with Deeleman-Reinhold (2001),Zonstein and Marusik (2020), Lin et al. (2022), Sankaran (2022), Lin et al. (2023) and Lu et al. (2023). Abbreviations : ALE: anterior lateral eye; AME: anterior median eye: CO, copulatory opening; DS, dorsal scutum; E, embolus; ES, epigastric scutum; RTA, retrolateral tibial apophysis; S, spermathecae; TA, tegular apophysis; VP, ventral process.

## Taxon treatments

### Oedignatha scrobiculata

Thorell, 1881

7A7B3336-03AF-5D63-97D8-8DC4FF2135A4

**Figure 2.**
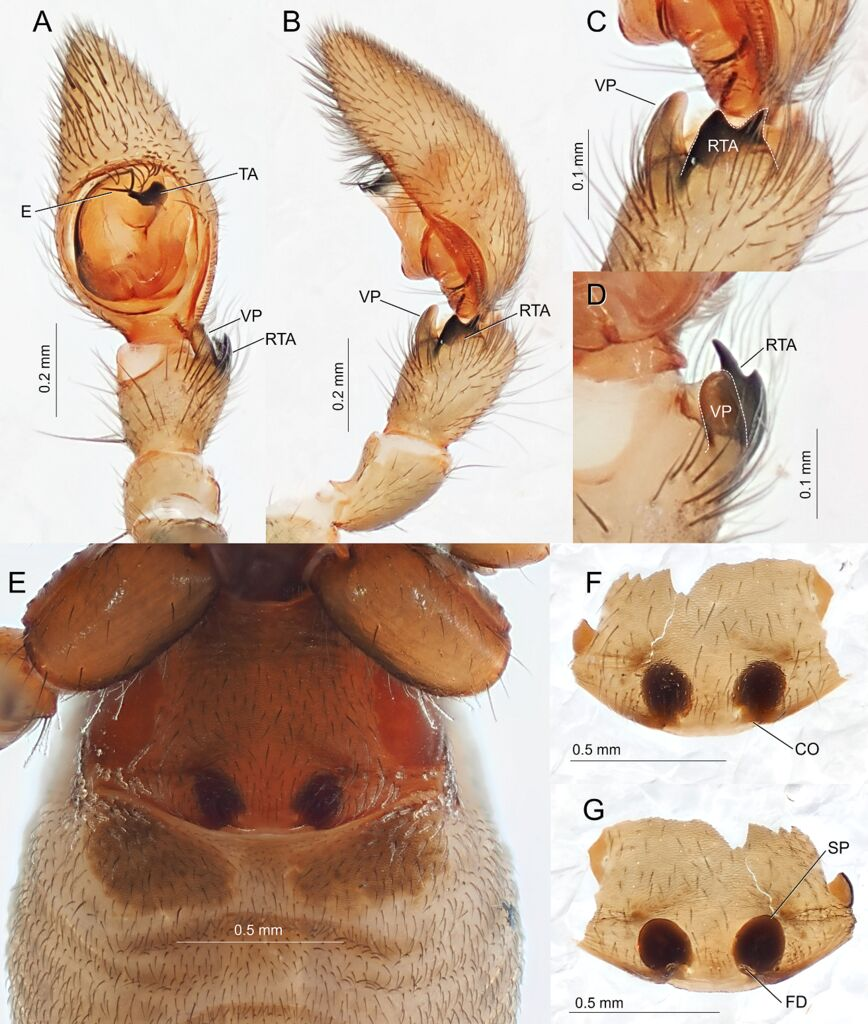
*Oedignathascrobiculata* Thorell, 1881; **A–D m**ale left palp (TKPM-AR 3191), ventral view (A), retrolateral view (B), magnified retrolateral view of retrolateral tibial apophysis (C), magnified ventral view of ventral process (D); **E–G** female genitalia (TKPM-AR 3193), ventral view of epigyne (E), ventral view of epigyne (dissected) (F), dorsal view of internal genitalia (G).

*Oedignathascrobiculata* Thorell, 1881 - Thorell (1881): 209, description of new species based on female holotype from Penang, Malaysia; specimen not examined); Gravely (1931): 268, figs. 18C–D (figures examined); Majumder and Tikader (1991): 116, figs. 240–245 (figures examined); Deeleman-Reinhold (2001): 267, figs. 348–356 (Synonyms of *Oedignathadecorata* and *Phrurolithusulopatulisus*; figures examined); Saaristo (2002): 9, figs. 18, 20 (figures examined); Tso et al. (2005): 46, figs. 1–3 (figures examined); Saaristo (2010): 61, figs. 6.8, 10 (figures examined); Jacquot et al. (2016) 323, figs. 2a–k (figures examined); Sankaran et al. (2019): 338, figs. 5A–E,H (Synonym of *Castianeirabengalensis*; figures examined); Mbo and Haddad (2020): 327, figs. 1C–F (Synonym of *Corinnanossibeensis*; figures examined); Caleb (2020): p. 15723, figs. 10A–F, 26G (figures examined); Koh et al. (2022): p. 194, 196, figs. of habitus and nest without numbers.
*Oedignathadecorata* Simon, 1897 - Simon (1897): 13 (description of female from Luzon; not examined)
*Corinnanossibeensis* Strand, 1907 - Strand (1907): 740 (description of female from Madagascar; not examined)
*Castianeirabengalensis* Biswas, 1984 - Biswas (1984): 120, figs. 4–6 (description of female from India; figures examined); Majumder and Tikader (1991): 141, figs. 297–301; Deeleman-Reinhold 2001: 396 (probably an *Oedignatha*).
*Phrurolithusulopatulisus* Barrion and Litsinger, 1995 - *Barrion and Litsinger (1995)*: 174, figs. 100a–i, 101a–e (description of male and female from Luzon; figures examined)

#### Materials

- **Type status:** Other material. **Occurrence:** recordedBy: Yu Hisasue; individualCount: 1; sex: male; lifeStage: adult; occurrenceID: 6A391A50-CCDE-5B7D-A984-2B37154B96EA; **Taxon:** scientificName: *Oedignathascrobiculata* Thorell, 1881; order: Araneae; family: Liocranidae; genus: Oedignatha; specificEpithet: *scrobiculata*; taxonRank: species; scientificNameAuthorship: T. Thorell; **Location:** islandGroup: Ogasawara Islands; island: Chichi-jima Island; country: Japan; stateProvince: Tokyo; county: Ogasawara-mura; municipality: Susaki; decimalLatitude: 27.0727; decimalLongitude: 142.1916; geodeticDatum: WGS84; **Identification:** identifiedBy: Yuya Suzuki; dateIdentified: 2024; **Event:** samplingProtocol: sifting; eventDate: 23-09-2023; **Record Level:** basisOfRecord: PreservedSpecimen
- **Type status:** Other material. **Occurrence:** recordedBy: Yu Hisasue; individualCount: 1; sex: female; lifeStage: adult; occurrenceID: 814E3C9F-BCD4-5633-B964-F6F72D7B4609; **Taxon:** scientificName: *Oedignathascrobiculata* Thorell, 1881; order: Araneae; family: Liocranidae; genus: Oedignatha; specificEpithet: *scrobiculata*; taxonRank: species; scientificNameAuthorship: T. Thorell; **Location:** islandGroup: Ogasawara Islands; island: Chichi-jima Island; country: Japan; stateProvince: Tokyo; county: Ogasawara-mura; municipality: Higashi-machi; locality: Mt. Ogami-yama; decimalLatitude: 27.0967; decimalLongitude: 142.1975; geodeticDatum: WGS84; **Identification:** identifiedBy: Yuya Suzuki; dateIdentified: 2024; **Event:** samplingProtocol: sifting; eventDate: 24-12-2023; **Record Level:** basisOfRecord: PreservedSpecimen

#### Description

**Male.** Measurements. Body 4.80 long; carapace 2.53 long, 1.60 wide, 1.06 high. Eye diameter: AME 0.14, ALE 0.14, PME 0.09, PLE 0.09. Eye interdistances: AME-AME 0.13, AME-ALE 0.07, PME-PME 0.19, PME-PLE 0.18. Length of legs: I, 1.60 + 0.55 + 1.63 + 1.51 + 0.92 = 6.21; II, 1.45 + 0.53 + 1.18 + 1.14 + 0.71 = 5.01; III, 1.10 + 0.43 + 0.77 + 1.01 + 0.53 = 3.84; IV, 1.70 + 0.58 + 1.56 + 1.74 + 0.69 = 6.27. Abdomen 2.36 long, 1.50 wide, 1.35 high.

Carapace longer than wide, pitted entirely. Clypeus with conical hump in front of AME. Chelicerae geniculate anteriorly, with pair of spines on dorsal-prolateral side. Palp (Fig. 2A–D): ventral process thumb-shaped, lightly pigmented; retrolateral tibial apophysis bifid, strongly pigmented, with dorsal apex sharper than ventral one; embolus long, filiform; tegular apophysis claw-shaped. Abdomen longer than wide. Anterior dorsum of abdomen sclerotised. Dorsum of abdomen entirely covered with scuta.

**Figure 3.**
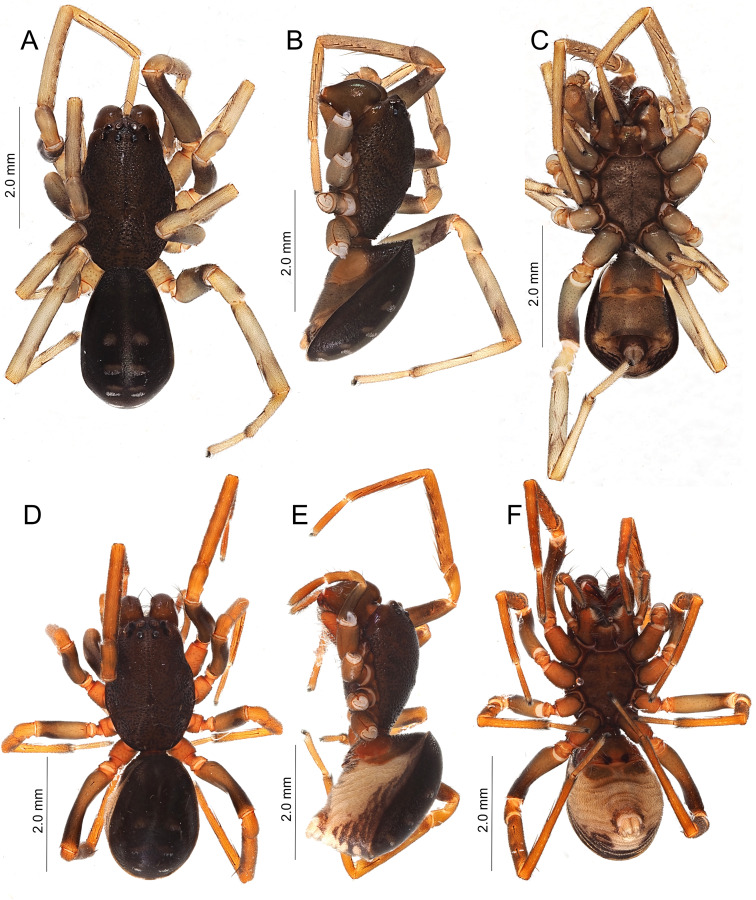
Habitus of *Oedignathascrobiculata* Thorell, 1881 from Chichi-jima Island, Japan. **A–C** male (TKPM-AR 3191); **D–F** female (TKPM-AR 3193); dorsal view (A, D); lateral view (left legs removed) (B, E); ventral view (C, F).

Colouration and markings (Fig. 3A-C): carapace dark brown; chelicerae, labium, maxilla and sternum dark yellowish-brown; palp yellowish-brown; coxa and femora of legs dark greyish-brown; trochanter, patella, tibia, metatarsi and tarsi yellowish-brown; abdominal scuta dark brown with four pairs of light spots; ventral side of abdomen dark yellowish-brown.

**Female.** Measurements. Body 4.46 long; carapace 2.14 long, 1.49 wide, 0.89 high. Eye diameter: AME 0.13, ALE 0.15, PME 0.10, PLE 0.10. Eye interdistances: AME-AME 0.10, AME-ALE 0.05, PME-PME 0.19, PME-PLE 0.13. Length of legs: I, 1.75 + 0.69 + 1.66 + 1.33 + 0.79 = 6.22; II, 1.31 + 0.49 + 1.08 + 1.06 + 0.61 = 4.55; III, 0.98 + 0.46 + 0.77 + 1.03 + 0.58 = 3.82; IV, 1.58 + 0.63 + 1.54 + 1.73 + 0.77 = 6.25. Abdomen 2.15 long, 1.52 wide, 1.63 high.

General appearance same as the male. Female genitalia (Fig. 2E–G): epigyne lacking membranous region; copulatory opening small, slit-like; spermathecae globular, separated; copulatory ducts indistinctive; fertilisation ducts short, curved.

**Figure 4.**
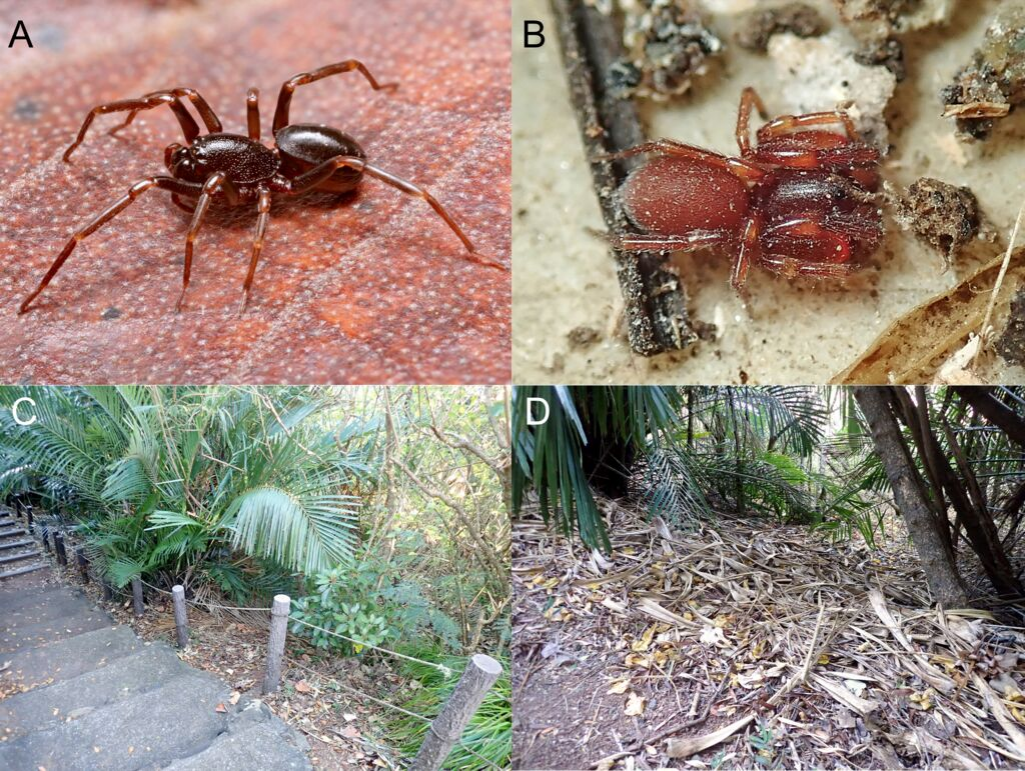
Live spiders and the habitats of *Oedignathascrobiculata* Thorell, 1881 and *Boagriusqiong* Lin & Li, 2022 on Chichi-jima Island, Japan. **A** female of *O.scrobiculata* (TKPM-AR 3193); **B** male of *Boagriusqiong* Lin & Li, 2022 (TKPM-AR 3219). **C, D** habitats at Mt. Ogami-yama, Chichi-jima Island.

Colouration and markings (Fig. 3D–F, Fig. 4A): same as the male.

#### Diagnosis

*Oedignathascrobiculata* can be distinguished from congeners by the combination of the following characteristics: bifid retrolateral tibial apophysis (Fig. 2C); blunt, finger-like ventral process of male palp (Fig. 2D); absence of membranous area of epigyne (Fig. 2E and F); internal genitalia lacking distinct copulatory ducts (Fig. 2G); presence of four pairs of light spots on the dorsal abdominal scutum (Fig. 3A, D and Fig. 4A).

#### Distribution

India, Thailand, Malaysia, Philippines, Indonesia, Taiwan. Introduced to Madagascar, Seychelles, Reunion, Germany. Probably introduced to Japan (Chichi-jima Island in the Ogasawara Islands, Tokyo: Fig. 1) (World Spider Catalog 2024, present study).

### Boagrius qiong

Lin & Li, 2022

A041CB6D-24EB-5C55-A5D6-AB933862516A

*Boagriusqiong* Lin et al., 2022 - Lin et al. (2022): 28, f. 1A–D, 2A–F (Male holotype (IZCAS-Ar 42724) from Xiuying District, Haikou, Hainan, China; photographs examined).

#### Materials

- **Type status:** Other material. **Occurrence:** recordedBy: Yu Hisasue; individualCount: 1; sex: male; lifeStage: adult; occurrenceID: 8339A370-8A92-573D-9431-EB02F53405AF; **Taxon:** scientificName: *Boagriusqiong* Lin & Li, 2022; order: Araneae; family: Palpimanidae; genus: Boagrius; specificEpithet: *qiong*; taxonRank: species; scientificNameAuthorship: YJ. Lin & SQ. Li; **Location:** islandGroup: Ogasawara Islands; island: Chichi-jima Island; country: Japan; stateProvince: Tokyo; county: Ogasawara-mura; municipality: Higashi-machi; locality: Mt. Ogami-yama; decimalLatitude: 27.0967; decimalLongitude: 142.1976; geodeticDatum: WGS84; **Identification:** identifiedBy: Yuya Suzuki; dateIdentified: 2024; **Event:** samplingProtocol: sifting; eventDate: 24-12-2023; **Record Level:** basisOfRecord: PreservedSpecimen

#### Description

**Male.** Measurements. Body 3.01 long; carapace 1.56 long, 1.17 wide, 0.90 high. Eye diameter: AME 0.10, ALE 0.05, PME 0.06, PLE 0.04. Eye interdistances: AME-AME 0.09, AME-ALE 0.07, PME-PME 0.17, PME-PLE 0.13. Length of legs: I, 0.96 + 0.67 + 0.62 + 0.30 + 0.34 = 2.89; II, 0.71 + 0.49 + 0.54 + 0.45 + 0.29 = 2.48; III, 0.73 + 0.37 + 0.53 + 0.51 + 0.33 = 2.47; IV, 1.04 + 0.45 + 0.78 + 0.71 + 0.35 = 3.33. Abdomen 1.61 long, 1.15 wide, 1.33 high.

**Figure 5.**
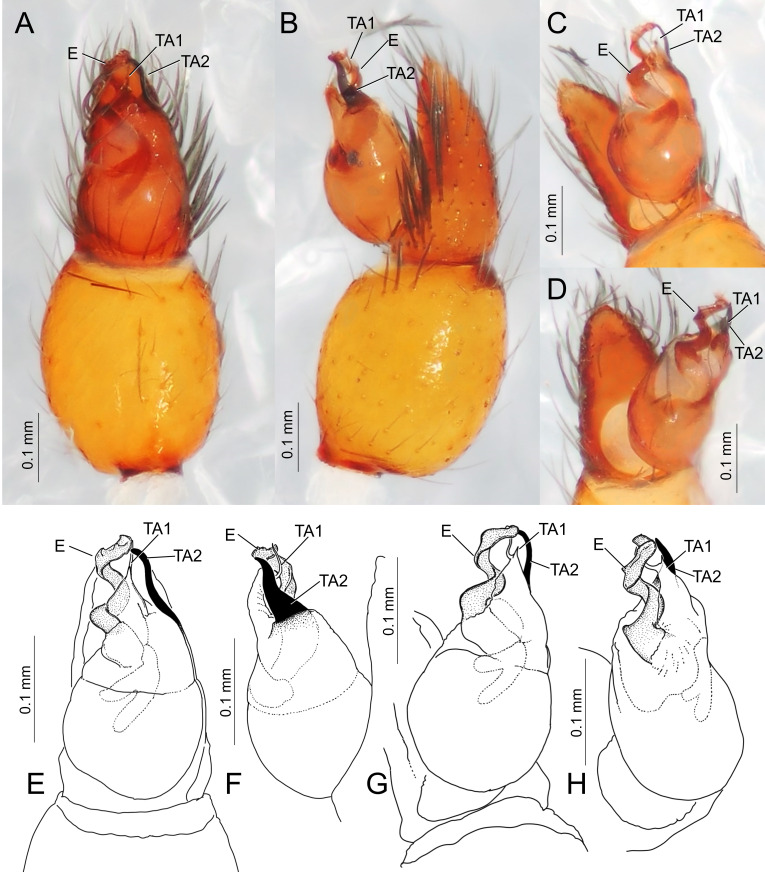
Male left palp of *Boagriusqiong* Lin & Li, 2022 from Chichi-jima Island, Japan (TKPM-AR 3219). **A, E** ventral view; **B, F** retrolateral view; **C, G** ventral-prolateral view; **D, H** prolateral view.

Carapace oval, slightly longer than wide (length/width 1.33), covered with small granules. Fovea short. ALE and PLE juxtaposed. Palp (Fig. 5): tibia barrel-shaped, embolus spiralled, tegular apophysis branched into two parts: TA1 with membranous triangular base and thin projection; TA2 robust, screlotised, triangular. Femur I robust; metatarsi I and tarsi I with well-developed prolateral scopula; metatarsi II–IV with distal preening brush. Abdomen oval, longer than wide (length/width 1.40). Anterior region of abdomen covered with dorsal and epigastric scuta.

**Figure 6.**
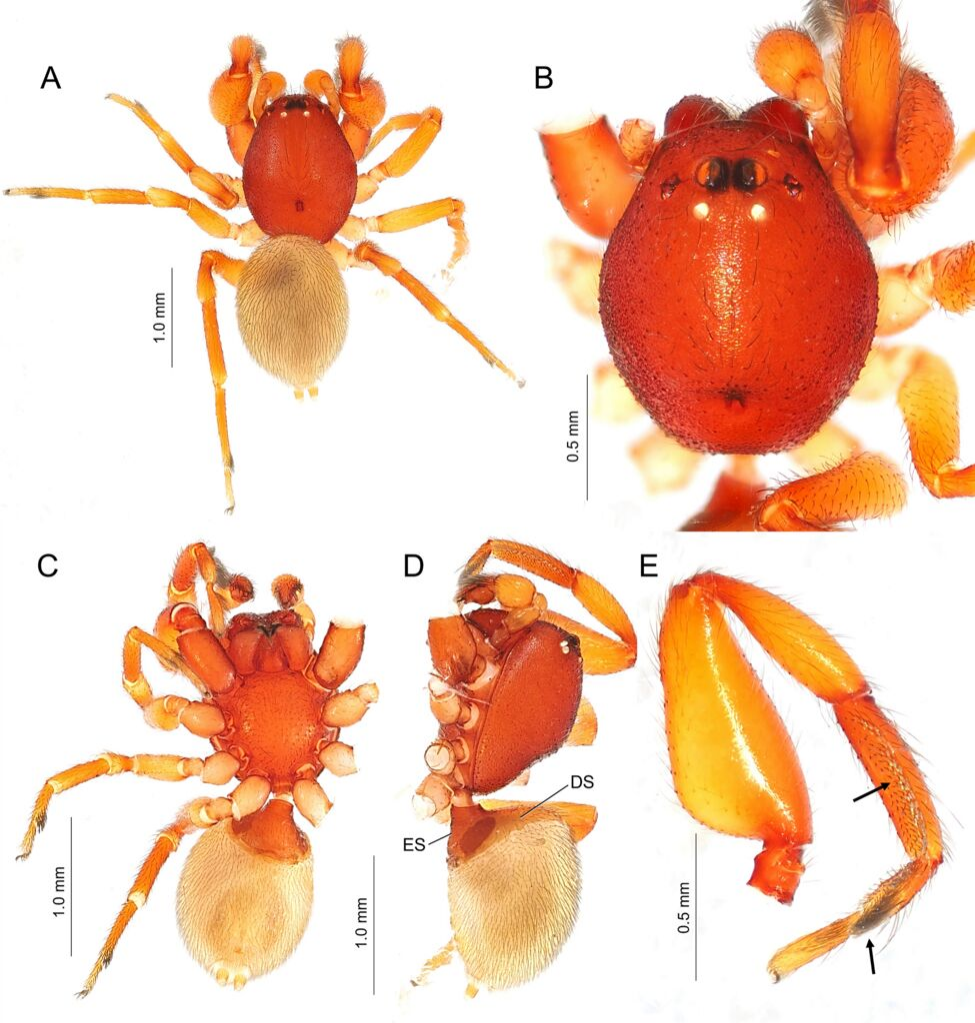
Male of *Boagriusqiong* Lin & Li, 2022 from Chichi-jima Island, Japan (TKPM-AR 3219). **A** habitus, dorsal view; **B** carapace, dorsal view; **C** habitus, ventral view; **D** habitus, lateral view, left legs removed; **E** left leg I, prolateral view. Arrows indicate prolateral scopula of tibia and distal preening brush of metatarsus.

Colouration and markings (Fig. 6 and Fig. 4B): carapace, chelicerae, maxillae, sternum and epigastric scutum reddish-orange, palps and legs yellowish-orange, abdomen reddish-orange in live spider, whereas turning to pale yellow in ethanol.

#### Diagnosis

*Boagriusqiong* resembles *B.tenuisus* Sankaran, 2022 in the general appearance and the palpal morphology, but can be distinguished by the shape of the tegular apophysis: TA2 (retrolateral branch) triangle-shaped, wide; TA1 (prolateral one) strongly curved, narrowed, with pointed apophysis on the middle region; TA2 slightly exceeding TA2 (Fig. 5) (cf. both TA1 and 2 narrowed, TA1 slightly curved, lacking pointed apophysis in the middle, TA2 clearly exceeding the tip of TA1 in *B.tenuisus*: Sankaran 2022, fig. 6)

#### Distribution

China (Hainan), Japan (Chichi-jima Island)

## Discussion

Our survey revealed the presence of two tropical ground-dwelling spiders, *Oedignathascrobiculata* and *Boagriusqiong* on Chichi-jima Island. These spiders represent novel additions to the spider fauna of the Ogasawara Islands and Japan. Amongst them, *O.scrobiculata* spiders were collected at various developmental stages, ranging from small juveniles (identified, based on the general appearance and excluded from the materials section) to adults. This suggests that the species have been reproducing and establishing a population on Chichi-jima Island. In contrast, we obtained only a single individual of *B.qiong* from the Island and it remains uncertain whether its population has been established.

Mbo and Haddad (2020) proposed that *O.scrobiculata* populations in Madagascar, Seychelles and Reunion may have been introduced from Asia, because most species of the genus inhabit South to Southeast Asia and none of the congeners has been discovered from the African continent. The habitat type of this species in Southeast Asia, artificially disturbed environments such as palm plantations (Deeleman-Reinhold 2001), further suggests that human activities may enable *O.scrobiculata* to expand its distribution range. Contrary to *O.scrobiculata* which exhibits a broad distribution, *B.pumilus* was recorded only from Hainan, China before the present study (Lin et al. 2022). Another three species of *Boagrius* are also known to inhabit relatively restricted regions: Malaysia to Sumatra (*B.pumilus*), Borneo (*B.simoni*) and India (*B.tenuisus*) (World Spider Catalog 2024). Consequently, this marks the first occurrence of *Boagrius* species on a small, isolated oceanic island in the Pacific Ocean.

On Chichi-jima Island, *O.scrobiculata* and *B.qiong* were collected from a litter layer beside a walkway on Mt. Ogami-yama (Fig. 4C and D), along with large numbers of several alien invertebrate species, such as *Porcellionidespruinosus* (Brandt, 1833) (Isopoda, Porcellionidae), *Technomyrmexbrunneus* Forel, 1895 (Insecta, Hymenoptera, Formicidae) and *Satsumamercatoriamercatoria* (L. Pfeiffer, 1845) (also recognised as *S.miyakoensis* M. Azuma & Y. Azuma, 1987; Kameda (2018)) (Gastropoda, Stylommatophora, Camaenidae). It is unclear whether these populations of invasive invertebrates were introduced simultaneously or prior to the two spider species into the site. Nevertheless, the site is considered to be susceptible to the invasion of invasive species. A similar situation was observed on Hachijo-jima Island in the Izu Islands, Tokyo, Japan. On this Island, a funnel web spider, *Coelotesoshimensis* (Araneae, Agelenidae), originally from Amami-oshima Island in the Nansei Islands, co-existed sympatrically with a large number of invasive arthropods, such as *Opisthoplatiaorientalis* (Blattaria, Blattodae) and *Chamberliniushualienensis* (Diplopoda, Polydesmida, Paradoxosomatidae) (Okumura and Cheng 2023).

Chichi-jima Island is approximately 2,000 km away from Taiwan and the Philippines and 3,400 km away from Hainan, where *O.scrobiculata* and *B.qiong* have, respectively, been recorded. Given this geographic distance, it is less likely that these ground-dwelling species reached Chichi-jima Island through natural dispersion, such as ballooning or current dispersion. Notably, both species were not collected on Chichi-jima Island during the field surveys conducted between 2009 and 2010 (Ono 2011, Ono, personal communication). Based on the above information, we hypothesise that the considered species, especially *O.scrobiculata*, are not native inhabitants of Chichi-jima Island, but were artificially transported to the Island, potentially through shipments of cargo containing seedlings and soil in recent years.

It is also important to consider the potential impact of the two spiders on the fauna of Chichi-jima Island. *Oedignathascrobiculata* is known for constructing silken tubular nests with multiple openings in the soil, ambushing prey from within (Koh et al. 2022). This behaviour indicates that *O.scrobiculata* is likely a predator of soil arthropods. Palpimanid spiders are recognised as araneophages, meaning they feed on other spiders and known to specifically prefer cursorial spiders (Pekár et al. 2022). These observations suggest a potential predatory influence of *O.scrobiculata* and *B.qiong* on other ground-dwelling arthropods in Chichi-jima Island. However, as mentioned earlier, the habitats of these two species on Chichi-jima Island are predominantly occupied by invasive ground-dwelling arthropods. This circumstance raises the possibility that prey-predator interactions may be established between alien prey and alien predators.

Our discovery further emphasises the significance of consistently monitoring arthropod fauna to promptly detect alien species. This proactive approach is also crucial for assessing the potential future dispersion of such species into other islands within the Ogasawara Islands and impact to other arthropods.

## Supplementary Material

XML Treatment for Oedignatha scrobiculata

XML Treatment for Boagrius qiong
